# Supplementary material for: Yiai Fuzheng decoction inhibits triple-negative breast cancer by remodeling the immune microenvironment
Source: Front Immunol. 2025 Sep 30;16:1615631. doi: 10.3389/fimmu.2025.1615631 (PMC12518410; doi:10.3389/fimmu.2025.1615631)
Supplement: Supplementary file 2 [file DataSheet1.zip › Supplementary File 2/Treatment/C_vs_D/07_Pathway_Analysis/Pathway_Analysis/Sig_Pathways_Name/Fructose and mannose metabolism.pdf]

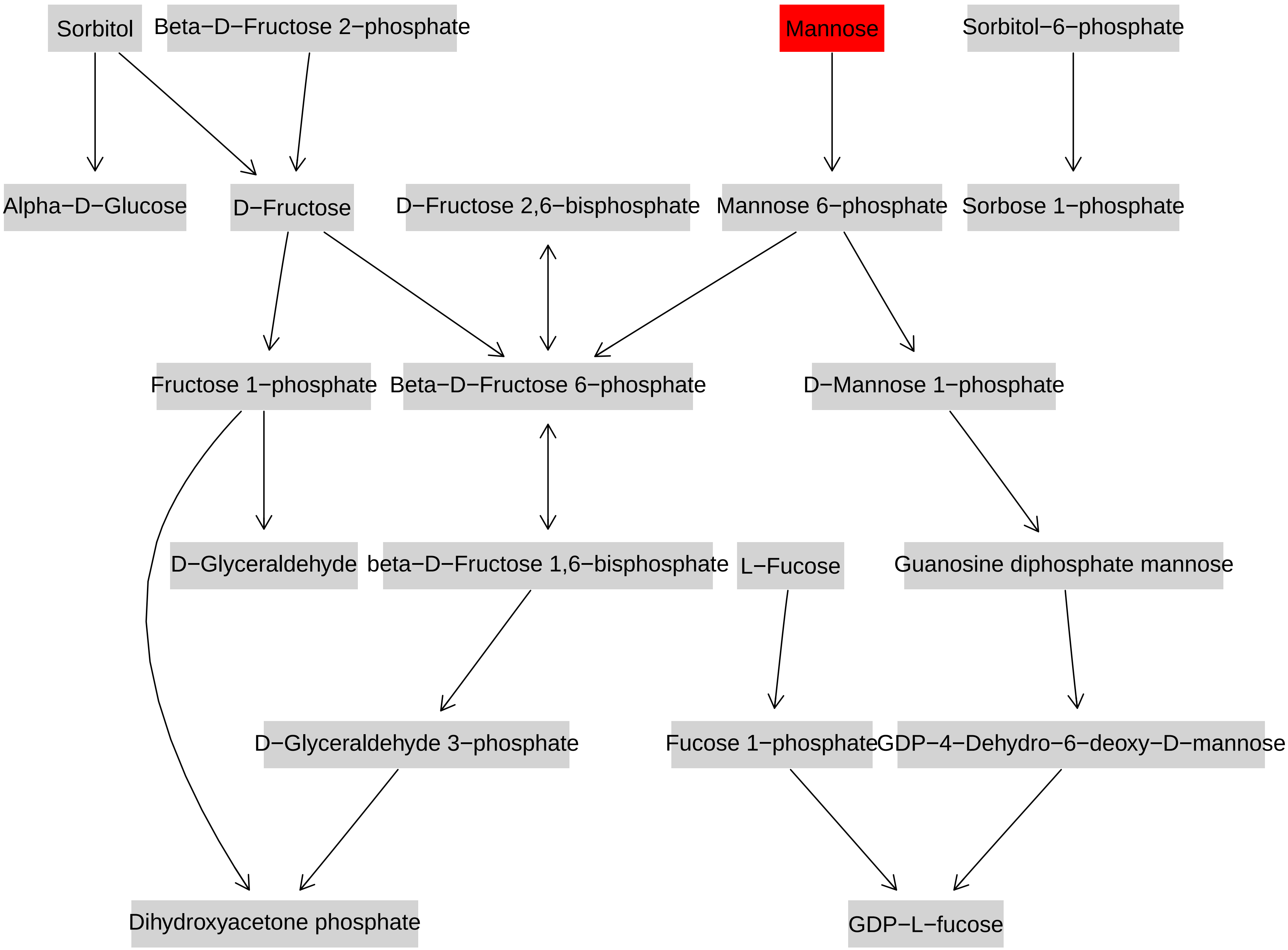
Sorbitol
Beta−D−Fructose 2−phosphate
Mannose
Sorbitol−6−phosphate
Alpha−D−Glucose
D−Fructose
D−Fructose 2,6−bisphosphate
Mannose 6−phosphate
Sorbose 1−phosphate
Fructose 1−phosphate
Beta−D−Fructose 6−phosphate
D−Mannose 1−phosphate
D−Glyceraldehyde
beta−D−Fructose 1,6−bisphosphate
L−Fucose
Guanosine diphosphate mannose
D−Glyceraldehyde 3−phosphate
Fucose 1−phosphate
GDP−4−Dehydro−6−deoxy−D−mannose
Dihydroxyacetone phosphate
GDP−L−fucose
